# Urea Inhibits the Formation of Environmentally Persistent Free Radicals as well as Accelerates Their Decay

**DOI:** 10.1021/acsomega.5c13197

**Published:** 2026-04-30

**Authors:** Divine Nde, Fox Foley, Orhan Kizilkaya, Myron Lard, Jennifer Richmond-Bryant, Lavrent Khachatryan, Slawomir Lomnicki, Phillip Sprunger, Stephania A. Cormier

**Affiliations:** † Department of Chemistry, 5779 Louisiana State University, Baton Rouge, Louisiana 70803, United States; ‡ Department of Physics and Astronomy, Louisiana State University, Baton Rouge, Louisiana 70803, United States; § Center for Advanced Microstructures and Devices, Louisiana State University, Baton Rouge, Louisiana 70806, United States; ∥ Department of Forestry and Environmental Resources, North Carolina State University, Raleigh, North Carolina 27695, United States; ⊥ Center for Geospatial Analytics, North Carolina State University, Raleigh, North Carolina 27695, United States; # Department of Environmental Sciences, Louisiana State University, Baton Rouge, Louisiana 70803, United States; ∇ Department of Biological Sciences, LSU Superfund Research Program and Pennington Biomedical Research Center, Baton Rouge, Louisiana 70808, United States

## Abstract

Environmentally persistent free radicals (EPFRs) have emerged as newly identified air pollutants, contributing to various environmental and health concerns. Consequently, the mitigation process, involving the prevention of their formation or acceleration of their decay, is pivotal in reducing the environmental risks associated with EPFRs. This study focuses on the mitigating effect of urea on the generation of EPFRs from 2-monochlorophenol (MCP) at 230 °C thermal remediation under laboratory conditions, abbreviated as MCP230 EPFRs. A strong inhibition of MCP230 EPFR formation was observed, increasing with the addition of urea to 10 wt % relative to the mass of 5% CuO/SiO_2_ particles prior to exposure. Loss of activity of Cu^2+^ toward EPFR formation in the presence of urea is likely due to the complexation of Cu^2+^ by urea or by ammonia, one of the major decomposition products of urea. Formation of this complex prevents the adsorption of EPFR precursor molecules on the surface-bound OH groups. It has also been observed that adding solid urea or a urea water solution accelerates the degradation of pre-existing EPFRs within the CuO/SiO_2_ solid matrix. In other words, a 1/e lifetime of MCP230 EPFRs from standard exposure was 42 h in a vacuum, while it decreased to 26 and 3 h when 5 and 10% urea were added, respectively, indicating that urea may reduce the stability of EPFRs. This hypothesis is also supported by characterization with X-ray spectroscopies. Our results suggest that urea possesses enormous potential for use as a mitigation agent for the prevention of EPFR formation and the acceleration of decay of existing EPFRs.

## Graphical abstract

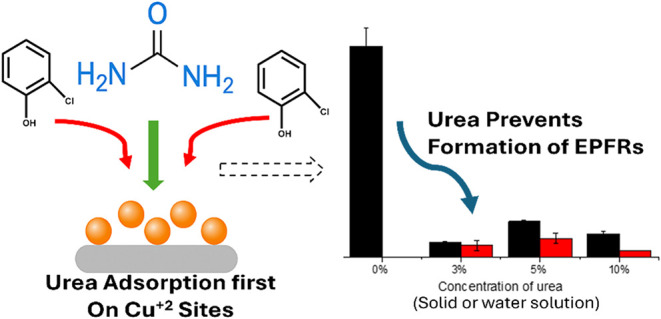

## 1 Introduction

Over the last two decades, significant concerns have arisen regarding environmentally persistent free radicals (EPFRs),^1–5^ due to their substantial potential to pose severe health hazards to biological and plant ecosystems.^6–8^ Toxicological responses from EPFR exposures have been widely demonstrated since 2000.^5,7,9–14^ Both *in vivo* and *in vitro* studies have indicated that individuals exposed to EPFRs risk developing pulmonary and cardiovascular diseases.^7,11,15,16^ EPFRs found in particulate matter (PM) smaller than 2.5 μm (PM_2.5_) contribute to DNA damage due to their capacity to generate reactive oxygen species (ROS),^17,18^ which cause oxidative stress responsible for the development and/or the promotion of heart and lung diseases.

Experimental and field studies have shown that EPFRs can remain in the environment for periods ranging from hours to months, depending on the type of EPFR and the environmental conditions.^19–24^ The type of EPFR formed also depends on the precursor molecules (e.g., benzene, monochlorophenol, and catechol),^21,23^ where EPFRs originate from combustion from different sources, such as thermal remediation of contaminated soils and sediments from Superfund sites and wildfires.^4,25–29^

Despite the large body of literature on health risks from EPFR exposures and the presence of EPFRs from combustion sources in the environment, there is a dearth of information on the mitigation of EPFR formation. We have focused our work on the interaction of model EPFR systems with chemical mitigators that can either prevent the formation of EPFRs on active surface sites or promote their quick degradation once formed. One such chemical mitigator is urea (NH_2_)_2_C=O, a nontoxic chemical used in many thermal treatment processes to reduce nitrogen oxide emissions.^30–33^ Urea has special ligating properties with transition metals^34,35^ either via oxygen^36^ or nitrogen^37^ (*vide infra*, Figure 6A–D), is readily soluble in aqueous solutions, and has long been used as a fertilizer or in the preparation of ammonium fertilizers.^38,39^

This study tested the hypothesis that urea inhibits the formation of EPFRs via complexation with the metallic core (in this case Cu^2+^). We test this hypothesis via laboratory experiments using laboratory-generated EPFRs formed at 230 °C from 2-monochlorophenol (MCP), referred to as MCP230 EPFRs. Additionally, we report the phenomenon of accelerated decay of EPFRs in the presence of urea, observed both for MCP230 EPFRs and for more complex EPFRs formed during the combustion of 1-methylnaphthalene (1-MN) in the presence of certain organics.^3^

## 2 Materials and Methods

### 2.1 Reagents

Pure 2-chlorophenol (MCP, 99%) was obtained from Sigma-Aldrich, Anthracene (99% purity), and urea (99–100% purity, powder; solid urea pellets Certified ACS grade, Catalog No. U15500, CAS no. 57-13-6, density 1.335 g/cm3, 99.0–100.5% purity) from Thermo Fisher Scientific, 1-methylnaphthalene (>96.0%) from TCI America and used without any further treatment. Cab-O-Sil, as silica powder, and a precursor for the preparation of 5% CuO (3.9% Cu) particles, copper nitrate hemipentahydrate (ACS reagent, 98%), were obtained from Cabot (EH-5, 99+ %) and Sigma-Aldrich, respectively. CuO nanoparticles (NP; 25–55 nm, stock no. US3063, CAS no. 1317-380-0, SSA 13.98 m2/g, 99.95% purity) were obtained from US Research Nanomaterials, Inc. and used as received as a reference.

Two types of EPFRs were generated and used in this work.

(i) **Gas-phase generation**: EPFRs were generated under exposure of a catalyst consisting of 5%CuO/SiO_2_ nanosized particulates to the vapor of the adsorbate, MCP, at 230 °C^1^ (Figure S1). The MCP solution was subjected to deaeration (degassing) from dissolved air by immersing the container in liquid nitrogen and evacuating the air under vacuum after a freezing/thawing cycle repeated three times. Typically, the 5%CuO/SiO_2_ powder undergoes drying at 120 °C within the exposure chamber (Figure S1) for 15 min, followed by a subsequent temperature rise to 230 °C. The 5%CuO/SiO_2_ catalyst underwent several cycles of exposure to saturated vapors of MCP at 230 °C under a vacuum of 10^–2^ Torr, with each exposure lasting 5 min. This process ensured surface saturation, as the EPR signal intensity stabilized after 5 repetition cycles. The exposure chamber was then cooled to 50 °C under vacuum, after which the particulate was analyzed by EPR.
(ii) **Combustion reactor generation**: The details of EPFR generation from a two-stage combustion reactor (TSCR) can be found in a recent reference^3^ and in Supporting Information (Figure S2). Briefly, EPFRs were synthesized through the catalytic combustion of 1-methylnaphthalene^40,41^ (1-MN) in the presence of anthracene and MCP, as a chlorinated organic representative. Specifically, this reactor provides an accurate control over EPFR concentration while allowing for the addition of other conditions specific to environmental samples,^25^ such as metal oxides (Fe_2_O_3_, CuO, NiO, etc.) at varying concentrations, in the reaction feed.  Iron oxide (III) nanoparticles (the catalyst) are generated from Zone 1 at 700 °C and transferred into Zone 2 (Figure S2). The fuel is delivered to Zone 2, and the products are formed catalytically in Zone 2 at 975 °C and collected thermophoretically onto a Cab-o-Sil fume silica matrix contained at the exit of Zone 2.

### 2.2 EPR Measurements

EPR measurements were conducted using a double cavity Bruker EMX-20/2.7 EPR spectrometer of X-band, 100 kHz, and microwave frequency of 9.76 GHz. Operating conditions for all measurements were 2 mW, 4 G, 200 G, 40.960 ms, and 167.7 s for microwave power, modulation amplitude, sweep width, time constant, and sweep time, respectively, using 1024 points with a receiver gain of 1.0 × 10^4^. WIN-EPR software was used to analyze the EPR spectra to measure *g* and Δ*H*_p–p_ values; the *g*-value is a representation of the splitting of energy levels resulting from electron spin interactions with the external magnetic field and is characteristic of each radical, while Δ*H*_p–p_ represents the peak-to-peak value for the EPR derivative spectrum in Gauss units.^42^ EPFR concentrations were calculated according to a standard procedure^42^ using the normalized double integration (DI/N) for both the sample and the reference, 2,2,-di­(4-tertoctylphenyl)-1-picryhydrazyl, (DPPH).^3^

Typical EPR spectra of samples generated either from the exposure chamber or the combustion reactor were characterized by relatively broad, unstructured peaks (Δ*H*_p–p_ ≈ 6–10 G), with *g*-values of 2.0038–2.0042 and 2.0028–2.0032, respectively.^3,43^ Preliminary analysis suggests that the complex spectra arise from a mixture of carbon- and oxygen-centered radicals (including soot radicals from the combustion reactor).^3,40,41^

### 2.3 Mitigation Studies with Urea

To evaluate the ability of urea to mitigate EPFRs generation and/or decay, two scenarios were evaluated: prevention of the generation of radicals and acceleration of EPFRs decay after formation. For prevention studies, urea was added to the sample or precursor before the generation of EPFRs as described in the preceding sections. For acceleration of decay, EPFRs were first generated before the addition of urea. Urea (10, 5, or 3% urea based on the mass of the catalyst) was crushed into fine powder with a mortar and pestle, and the calculated amount was added and mixed with the catalyst (for prevention of formation of EPFRs) or with the generated EPFRs (for acceleration of decay). After addition, the sample was incubated for 6 h and then subjected to EPR analysis. The EPR measurements were performed after thoroughly mixing and vortexing the solid samples. To address environmental relevance, a urea solution was also added to select the samples of interest.

### 2.4 X-ray Spectroscopies

To characterize interactions between urea and the catalyst, X-ray absorption near-edge structure (XANES) and X-ray photoelectron spectroscopy (XPS) were used. Samples of catalyst (5% CuO/SiO_2_) and cat-urea (catalyst mixed with 10% urea) were generated. For urea and MCP gas-phase exposure in XPS measurements, solid urea pellets were mechanically ground into lamellar fragments. These fragments were loaded into a glass dosing vial and connected to a standard leak valve on a custom dosing chamber (base pressure 10^–9^ Torr). Additionally, the organic precursor of liquid 2-chlorophenol was loaded into an identical vial. Both substances were purified through multiple freeze–pump–thaw cycles.

XANES measurements, which reveal oxidation states and electronic configurations of elemental components, were performed using synchrotron radiation at the variable-line-spaced plane grating monochromator (VLSPGM) beamline at Louisiana State University’s Center for Advanced Microstructures and Devices (CAMD) in Baton Rouge, Louisiana. Powder samples were prepared by depositing onto carbon tape adhered to a stainless-steel sample holder and then inserted via load/lock into the chamber maintained at a baseline pressure of ∼10^–9^ Torr. Urea fragments were prepared by drop-casting a slurry of urea and ethanol onto a Au-coated silicon wafer attached to a sample holder. After ethanol evaporation, the urea sample was loaded into the ultrahigh vacuum system. Spectra were acquired in total electron yield mode at room temperature, with multiple scans per sample to improve data quality and signal-to-noise ratio. CuO NP served as a reference for monochromator calibration, utilizing the Cu L-edge at a photon energy of 935.12 eV. Data analysis was performed using Demeter’s Athena 0.9.26 software, including spectral alignment, normalization, merging, and truncation. Final processing and visualization of data were performed using IGOR Pro 9.05.

XPS, which is more surface-sensitive than XANES, was also used to examine the effects of urea on the MCP230 EPFRs. Measurements were carried out on a modified Scienta ESCA system equipped with an EA 128 hemispherical analyzer and a dual-anode XM1200 source (Al K_α_/K_β_), with a base pressure in the analysis chamber of approximately 1 × 10^–10^ Torr. Since heating is required for dosing organic vapors, carbon tape was not used. Instead, all XPS samples were prepared by drop-casting, aligning with the preparation method for urea fragments used in XANES, creating a thin, uniform layer on a Au-coated silicon substrate, mounted on a Ta platen. Samples were loaded into the system via load/lock and transferred into the analysis chamber. For gas-phase dosing, a custom chamber with an open filament for heating, a retractable thermocouple probe, and standard leak valve attachments (for urea and MCP vials) was attached to the XPS system to avoid oxygen contamination and maintain low pressure during measurements. Spectral processing, including background subtraction and peak fitting, was performed using Casa XPS software.^44^ Since all samples were on a Au substrate, the Au 4f_5/2_ peak was calibrated to 87.63 eV. Final data visualization was conducted using IGOR Pro 9.05.

## 3 Results and Discussion

### 3.1 Interactions between Urea and 5%CuO/SiO_2_ Catalyst

To better understand the chemical interaction between the resulting urea and MCP230 EPFRs, we first focus on elucidating details of the catalyst-urea (cat-urea) interaction. Figure 1 shows XANES (a, b) and XPS (c) spectra illustrating urea binding to the catalyst. The N K-edge spectra were calibrated using boron nitride (BN) as a reference (white line = 402.5 eV). The N K-edge (Figure 1a) of pure urea exhibits a sharp N 1s → π* transition peak at 403.8 eV from the amine group NH_2_ and a prominent 1s → σ* peak at 405.7 eV, along with another 1s → σ* contribution forming a broad peak around ∼413.5 eV. This aligns with previous observations of other amino acids.^45,46^ Cat-urea shows a spectrum very similar to that of urea but lacks both a shoulder on the σ* peak at 405.7 eV and the broad peak near 413 eV. Cat-urea also displays a small N 1s → π* peak at 399.3 eV, absent in the urea spectrum, which suggests an oxidized NH_*x*_ species and confirms urea bonding to the catalyst through nitrogen (N). XPS (Figure 1c) N 1s measurements support this, with urea showing a single amine peak at 399.5 eV. As shown, fitting of the nitrogen cat-urea exhibits the same peak shifted by +0.6 eV between “Peak 0” and “Peak 1” toward higher binding energy (BE). The overlapping, broader peak at 404.5 eV is a contribution from Ta 4p peaks of the sample platen and can be disregarded. As in the XANES spectra, the shift of cat-urea’s N 1s peak again suggests formed NH_*x*_ species, now in an oxidized state. Again, these data suggest that urea bonds primarily to the catalyst through N atoms. Evidence of carbonyl loss during urea binding is also seen in the C K-edge spectrum of cat-urea (Figure 1b), where the C=O peak is 92% less intense than in urea. This is further supported by the XPS C 1s spectrum (Figure S3d). O spectra (Figure S3a) are more complex because of the many different oxygen species present, making peak assignment and contribution identification difficult, even with CuO and urea standards. Therefore, we focus on a calculated difference spectrum obtained by subtracting the cat-urea spectrum from that of the catalyst. The peak at 533 eV is likely mainly from the underlying SiO_2_, as the major CuO peak at ∼531 eV is not seen in either sample, though it appears larger in cat-urea due to urea contributions. The peak at ∼537 eV is also primarily from SiO_2_. O 1s XPS spectra (Figure S3c) support this, showing increasing O content in the order urea > catalyst > cat-urea. The metal Cu L-edge (Figure S3b) was analyzed using CuO nanoparticles as a reference. Both the catalyst and catalytic urea show a weak but identifiable Cu L-edge signal, with peaks at the same energy but shifted by −0.9 eV relative to CuO, likely due to interactions with SiO_2_. The 33% intensity attenuation of the Cu L-edge signal in cat-urea suggests the catalyst is reduced when mixed with urea.

**Figure 1:**
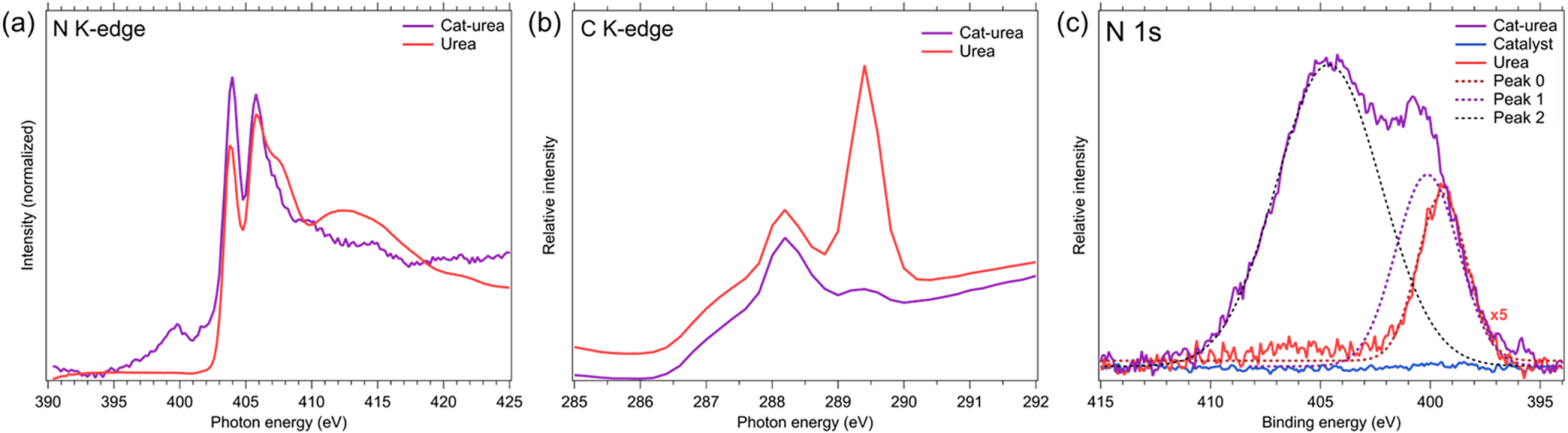
(a) XANES N K-edge of cat-urea and urea; peaks above 402 eV are attributed to σ-bonds. (b) C K-edge of urea and cat-urea. The cat-urea peak at 289.5 eV is attenuated. (c) XPS N 1s spectra of undosed catalyst (no N), urea, which has a single peak at 399.5 eV BE, and cat-urea exhibiting a positive BE shift in its main N 1s peak by 0.6 eV. The peak at 404.5 eV is from Ta-4p_3/2_ sample holder contamination.

In summary, the appearance of a new peak and visible changes in the nitrogen K-edge peak intensity, along with the reduction in the carbonyl-related feature in both the carbon K-edge and the C 1s XPS spectra, suggest chemical changes consistent with partial urea decomposition. Specifically, the emergence of an additional nitrogen-related feature and the new peak in the nitrogen 1s spectrum shifted by 0.6 eV compared to that of intact urea indicate the formation of new nitrogen-containing species, possibly from the breakdown of urea’s functional groups. At the same time, the reduced carbonyl signal strength points to a loss or transformation of the carbonyl groups, supporting this interpretation. Overall, these spectral changes imply alterations in the local electronic structure and bonding, which can reasonably be attributed to thermal or chemical decomposition of urea under the experimental conditions. Overall, the CuO/SiO_2_ catalyst matrix appears to be modified in the presence of urea in a manner that is most likely unfavorable for the formation of EPFRs, as observed experimentally (see Section 3.2).

### 3.2 The Prevention of Formation of MCP230 EPFRs by Urea

The influence of urea on the formation of EPFRs (Figure 2) is shown by laboratory adsorption studies conducted on the model system MCP. Urea was added to the catalyst of 5%CuO/SiO_2_ at concentrations of 5 and 10% w and subjected to exposure to saturated vapor of MCP in a vacuum at 230 °C (Figure 2A). A dramatic reduction of the concentration of EPFRs (∼92%) compared to the control (Figure 2A, blue curve) was observed when the initial mixture contained 10% (w) urea. In other words, the 1/e lifetime of MCP230 EPFRs under standard exposure is approximately 42 h in a vacuum, with the initial slow decay period lasting for the first 3–4 h. However, with the addition of 5 and 10% urea, the lifetime decreases to 26 and 3 h, respectively, illustrating the destabilizing effect of urea on EPFRs. Note that the 1/e lifetime of EPFRs can vary significantly depending on the quality of the vacuum within the ampule housing the EPFRs^43^ (Supporting Information, Section 3.1).

**Figure 2:**
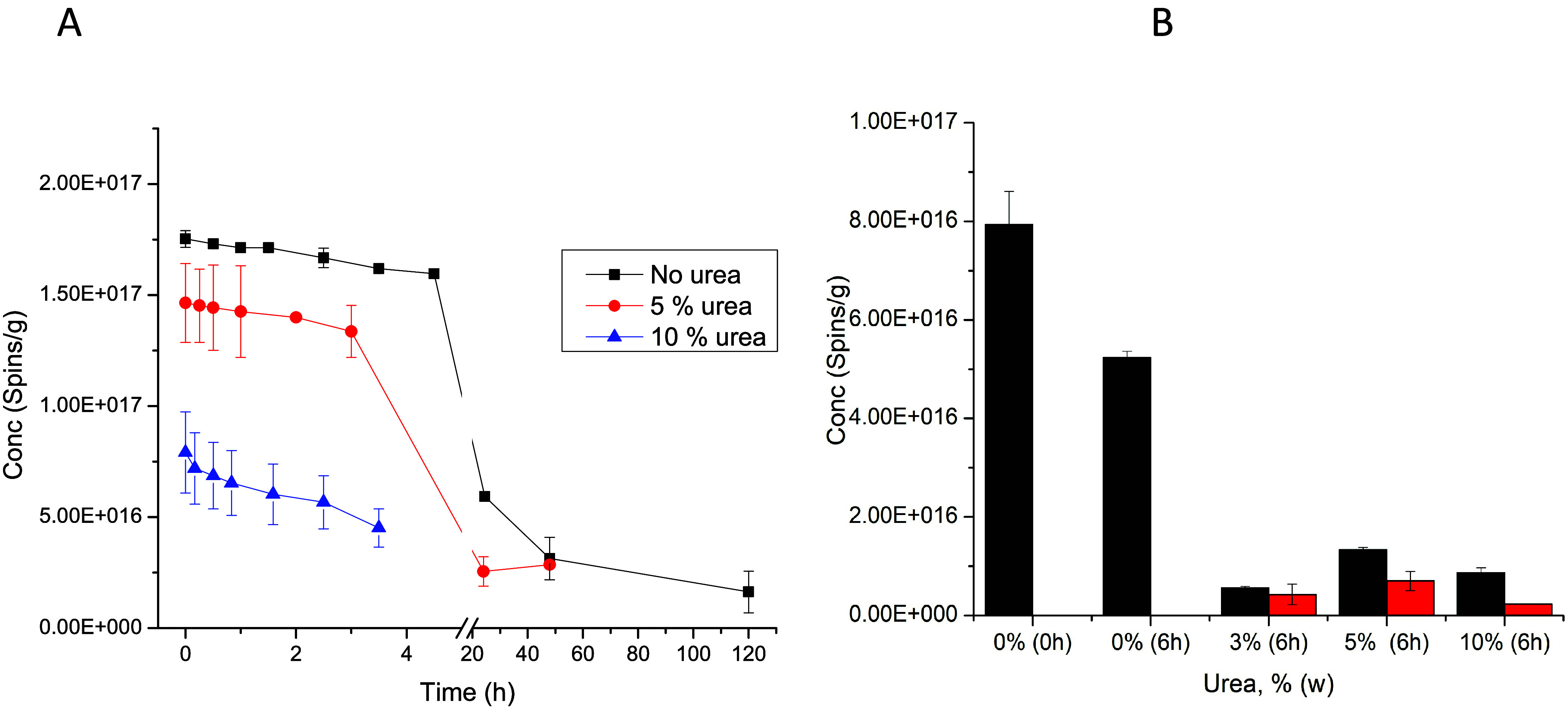
(A) Decay curves of MCP230 EPFRs as a function of urea concentration added beforehand to the catalyst CuO/SiO_2_ and subjected subsequently to exposure of MCP at 230 C. (B) Different percentiles of solid urea were added to pre-existing MCP230 EPFRs in powder status (black graph) and left under hood for 6 h without heating. Similarly, the same amount of urea in water solution was added to solid, pre-existing MCP230 EPFRs (red graphs) and left under the hood for 6 h.

### 3.3 Urea Accelerates the Degradation of Both Pre-existing MCP230 EPFRs and EPFRs Generated from Combustion Reactor

#### 3.3.1 Solid Urea Added to the Sample Containing MCP230 EPFRs

An addition of solid powdered urea to the already formed EPFRs has been examined (Figure 2B). The MCP230 EPFR concentration decreased by approximately 20% after 6 h of aging in the absence of urea (Figure 2B, second black graph). Urea at a concentration of 3–10% had a profound effect on the reduction of EPFRs concentration generated beforehand from MCP. The addition of 3–10% (w) urea to the EPFR-containing matrix resulted in a substantial reduction of more than 80% (Figure 2B, third-5th black graphs).

#### 3.3.2 Urea Dissolved in Water Added to the Sample Containing MCP230 EPFRs

The addition of urea dissolved in water to the sample containing EPFRs presents an environmentally realistic scenario. Significant reductions in the quantities of EPFRs were observed for the MCP230 EPFRs by soaking the sample in a water solution containing 3–10% urea (Figure 2B, red graphs). To characterize the effect of urea alone, a parallel sample was prepared containing only the generated EPFRs soaked in deionized water. The reduction of the concentration of EPFRs in deionized water (DI, an EPFR abatement of 80%) (Figure S5A) was equally effective compared to the experiment in which only solid urea was added (Figure S5B, an EPFR abatement of 82%), indicating that water also plays an important role in accelerating the decay of already formed EPFRs. However, there was a significant reduction with application of aqueous urea (Figure 2B, red graphs and Figure S5A, the final graph, an EPFR abatement of 97%), thereby confirming that the presence of water and urea could have a synergistic effect on the reduction of already formed EPFRs (*vide infra*, Section 3.7).

### 3.4 Interplay of MCP and Urea at the Surface

XPS supplements the EPR observations of MCP230 EPFR concentration (spins/g) following interaction with urea, and XPS provides insight into the mechanism of this codeposition interaction. To replicate mitigation strategies tested with EPR, XPS experiments involving both MCP and urea vapor were performed on catalyst and cat-urea samples. Catalyst samples dosed first with MCP then with urea mimic a post-MCP230 EPFR remediation strategy to achieve decay of EPFRs, while cat-urea samples dosed with MCP represent a preventative approach to using urea in MCP230 EPFR formation (Figure 3).

**Figure 3:**
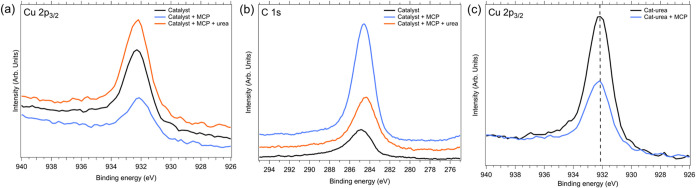
XPS spectra detailing remediative (a, b) and preventative (c) effects of urea vs MCP230. (a) Cu 2p_3/2_ shows MCP-EPFR formation reduction and subsequent elimination upon urea adsorption (b) C 1s peaks also reveal initial MCP adsorption and then attenuation with urea (c) Cu 2p_3/2_ peak of cat-urea is unshifted after organic dosing (dotted line at 932.17 eV), indicating urea prevents MCP from transferring charge to the surface.

The Cu 2p_3/2_ XPS spectrum of the undosed Cu/SiO_2_ catalyst (Figure 3a) shows a primary peak at 932.2 eV. After exposing it to ∼1 kL of MCP230 and holding it at 230 °C, XPS reveals a small (∼0.3 eV) shift to lower binding energy, indicating a reduction of copper. Previous XPS and XAS studies^47–49^ have linked this charge transfer from MCP to copper with the formation of EPFRs. However, when this system is subsequently exposed to 100 L of urea at room temperature, the Cu 2p_3/2_ peak returns to its original value of 932.2 eV (Figure 3a). This strongly suggests that urea either replaces or removes the adsorbed MCP230 EPFR. Furthermore, C 1s spectra of the catalyst show the expected adventitious carbon at 284.7 eV (Figure 3b), but with MCP230 addition, there is a substantial increase in carbon content and a slight positive shift in the peak to 284.2 eV, consistent with surface C=C aromatic ring bonds from adsorbed MCP. However, further urea adsorption on the catalyst + MCP results in a marked decrease in surface carbon intensity. This also supports the idea that urea helps remediate EPFRs via adsorption. XPS spectra of the Cu 2p_3/2_ peak exhibit no shift in energy after MCP230 dosing of the cat-urea (Figure 3c). This demonstrates that the presence of urea can prevent the charge transfer that is vital to the formation of MCP230-EFPRs.

The XPS results demonstrate the successful deposition of MCP230 and subsequent room-temperature urea vapor dosing, which markedly alters the bonds on the MCP230 and catalyst surfaces. This confirms the paper’s hypothesis that urea can inhibit EPFRs formation.

### 3.5 Urea Accelerates the Decay of EPFRs Generated from Combustion Reactors

The two-stage combustion reactor (TSCR; Figure S2) generates EPFRs from the combustion of 1-methylnaphthalene (1-MN) in the presence of additives 2-monochlorophenol + anthracene, with a well-defined and controlled, but complex, EPFR composition. The character of EPFRs was reported in our early^40,41^ and recent publications.^3^ The EPFRs were found to be a complex mixture of organic carbon-centered radicals, oxygen-centered radicals, and soot. In this case, urea dissolved in water was applied to the solid sample collected from the combustion reactor (Supporting Information, Figure S2). To comprehend the effect of urea alone, a control sample was prepared by adding deionized water to the generated EPFRs in the solid state (similarly to the case with MCP230 EPFRs) (Figure 2B). Substantial reductions in the quantities of EPFRs by 10% urea were observed for the complex EPFRs (Figure S5B), indicating that urea accelerates the decay of already formed complex EPFRs from the combustion reactor, as well.

#### 3.5.1 Remediation of the Field Sample(s)

Thus, the application of urea (especially in a water solution) to the deposited waste sites may play a critical role in accelerating the decay of already formed radicals. Therefore, the treatment of the contaminated sites with urea solution will likely play a dual role in accelerating the decay of existing radicals in the mixture (Figure S6, black line) while simultaneously preventing or limiting the generation of new radicals. This phenomenon has been tested on the field sample of soils obtained from properties within 10 miles of the Colfax, LA thermal remediation site, in which EPFRs were measured^50^ (Supporting Information, Section 3.2.1). A drop of EPR signal intensity of ∼20 to 25% was detected following the impregnation of the Colfax sample (abbreviated 2B) into a 10% urea solution (Figure S6, red line).

### 3.6 Characterization of Paramagnetic Species Formed during MCP230 EPFR Generation in the Presence of Urea

A nonfeatured, singlet spectrum of MCP230 EPFRs was detected in the presence of urea in the matrix (Figure S7A, Spectrum 1). The intensity of spectrum 1 with a *g*-value of 2.0040 (oxygen-centered radicals) drops after 2 days of aging to an apparent *g*-value of 2.0034, indicating a mix of oxygen-centered and carbon-centered radicals (Figure S7A, Spectrum 2). Similar trends in the change of *g*-value during the aging process are typical for laboratory-generated MCP230 EPFRs in the absence of urea^43^ (Supporting Information, Section 3.1 and Figure S4). The major observation is the noticeable detection of a Cu^2+^ EPR spectrum, while unresolved, in the presence of urea (Figure S7A, Spectrum 2) at *g*_iso_ (Cu^2+^) = 2.0725. A detectable decrease of *g*-value and Δ*H*_p–p_ value for the organic signal has been observed in the presence of urea, indicating a change in the local microenvironment of the radical with aging (Figure S8).

#### 3.6.1 Urea Increases the Resolution of the EPR Spectrum of Cu^2+^

Cat-urea (10%, w) mixture was thermally treated in the temperature region 110–250 C under vacuum following EPR examination (Figure 4). A weak spectrum appears at 110 °C (Spectrum 1) and slowly strengthens with temperature up to 250 °C (Spectrum 4). Then the sample was kept in the dark, and a continuous growth of the EPR spectrum of Cu^2+^ was detected (Figure S7B) until the intensity stabilized, showing a more well-defined feature 2 weeks later (Spectrum 5, Figure 4). The spectrum intensity of Cu^2+^ significantly rises when the urea concentration is increased from 5% (red curve) to 10% (black curve) (Figure S7B). In other words, the higher the urea content in the matrix, the stronger the Cu^2+^ EPR signal becomes.

**Figure 4:**
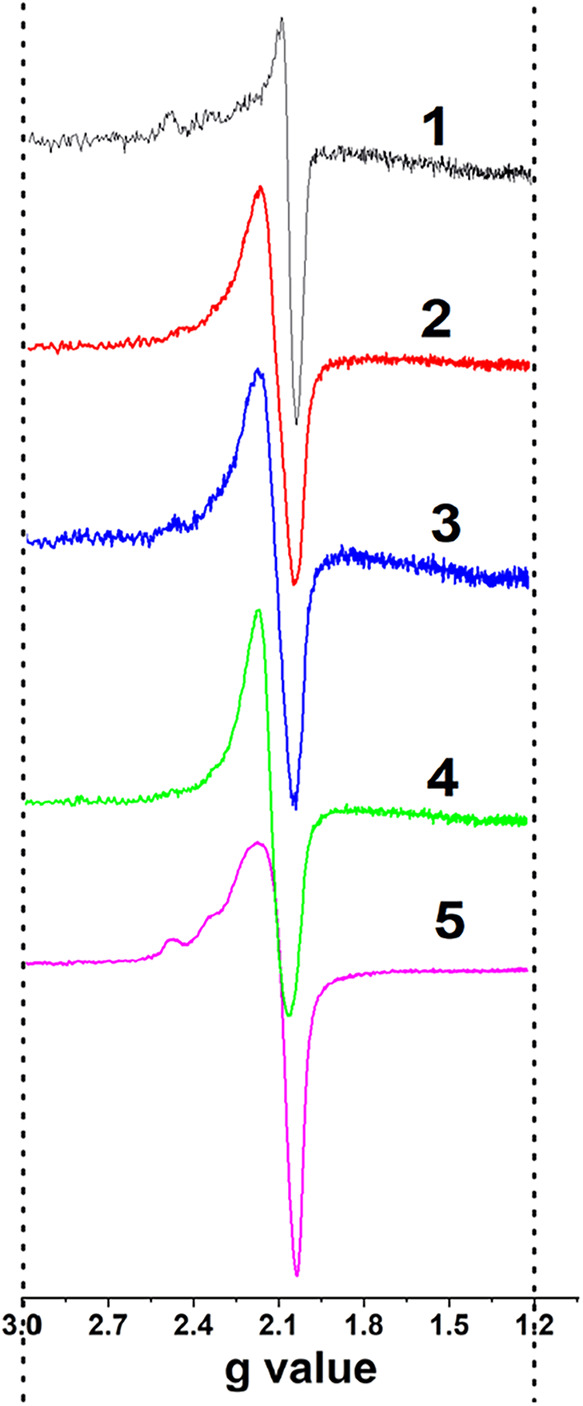
Evolution of the EPR spectrum of Cu^2+^ after heat treatment of a CuO/SiO_2_/urea (10 wt %) mixture at: 1–110, 2–205, 3–230, 4–250 °C (total measurement time: 75 min); 5 – spectrum recorded 2 weeks later.

Spectrum 5, while still not yet well resolved, shows some hyperfine splitting characteristics for axial symmetry (most probably disordered) of Cu^2+^.^51^ At nuclear spin of 3/2 for ^63^Cu^2+^ the Zeeman line will be split into four lines (*m*_I_ = 3/2, 1/2, −1/2, −3/2) and four equidistance peaks are expected.^52^ The EPR spectra of Cu^2+^ in different environments, phases, and matrices are well-known.^53–55^

The appearance of the EPR experimental signal for Cu^2+^ (Figure 4, Spectrum 5) indirectly indicates the occurrence of an interaction of urea molecules with the Cu^2+^ clusters. It is worth noting that nitrogen-containing products formed during high-temperature decomposition of urea (ammonia, isocyanic acid, hydrazine, etc.)—particularly ammonia—may exert a similar effect through complexation with copper active sites (*vide infra*, Section 3.8).

The Cu^2+^ (Figure 4, spectra 1–4) portion of the spectrum is not well resolved, probably due to the high content of Cu^2+^ in the catalyst (3.5 wt %) and the close vicinity of Cu^2+^ cations to each other, which leads to increasing dipole–dipole interaction^52,56^ and high concentration broadening (compare with Figure S7A). However, the experimental parameters for the hyperfine splitting constant (*A*_II_) values for a reasonably resolved spectrum of Cu^2+^ (Figure 5, spectrum (A), which is spectrum 5 in Figure 4), typical for axial symmetry^56^ of Cu^2+^, were extracted as *A*_II_ = 165 G (462 MHz) at *g*_II_ = 2.2913 and *g*_⊥_ = 2.0566. To validate these results, the EasySpin computational package^57^ was employed to simulate and analyze complex EPR spectra (Figure 5A). The EasySpin code (Supporting Information, Section 4) for simulation of Cu^2+^ EPR spectrum at low symmetry^45^ and axial g tensors extracted from the experiment (*g*_*x*_ = *g*_*y*_ = 2.0566, *g*_*z*_ = 2.2913 and *A*_II_ = 462 MHz) has been applied, and the results are compared in Figure 5B (blue line—experimental, red line—simulation). Therefore, very close proximity of experimental and simulated spectra (Figure 5B), as well as detailed XPS and XAS analysis of the cat-urea system and the MCP230 EPFR/catalyst system (with and without urea) supports the ligation theory of urea with Cu^2+^ within the CuO/SiO_2_ matrix. This conclusion leads us to hypothesize a mechanism of prevention for the generation of MCP230 EPFRs in the presence of urea (section below).

**Figure 5:**
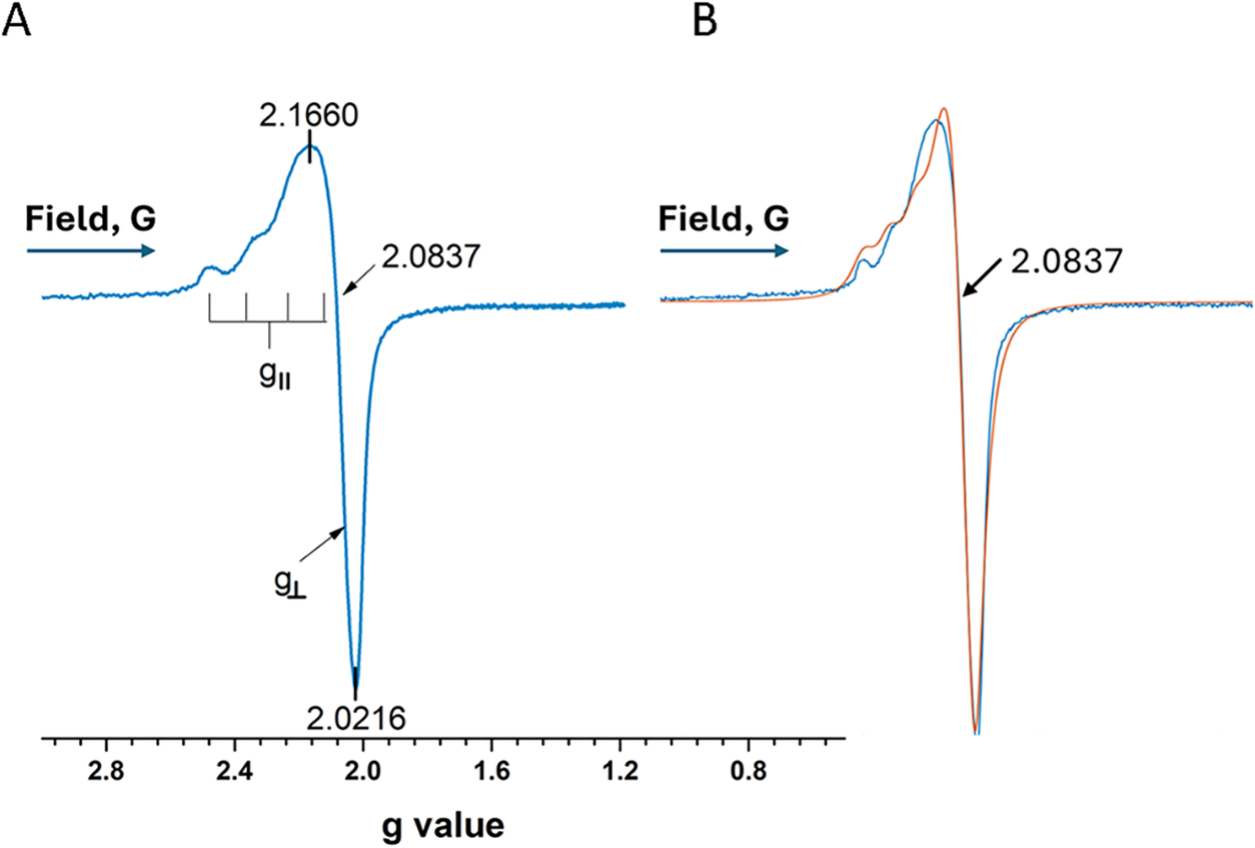
Experimental parameters of Cu^+2^ complexed with urea after heat treatment of CuO/SiO_2_ + 10% (w) urea: (A) *g*_⊥_ = 2.0566; *g*_II_ = 2.2913, *g*_iso_ for Cu^+2^ at baseline crossing with spectrum is 2.0837. Red line in (B) is an EasySpin simulation of the spectrum.

### 3.7 Proposed Mechanism for the Prevention of Formation of MCP230 EPFRs

To propose a mechanism for the prevention of EPFRs generation from simple precursors, it is useful to look at the conventional mechanism for the formation of EPFRs as explained by Dellinger and co-workers^1,16,58–60^ (Supporting Information, Section 5 and Scheme S1). Briefly, the mechanism postulates the removal of water molecules from the hydroxylated surface of the catalyst with subsequent electron transfer from the oxygen to the copper atom, reducing Cu (II) to Cu (I) while forming an oxygen-centered EPFR (Scheme S1). This oxygen-centered radical can then be resonance stabilized by forming a counterpart that is centered on carbon (Scheme S1). This conventional process of formation of EPFRs can be disturbed in the presence of urea. Indeed, urea and its principal decomposition products, ammonia (NH_3_) or isocyanic acid (HNCO),^33^ have special ligating properties with transition metals^34,35,37^ and can easily enter coordinate bonding with the Cu (II) portion of the catalyst. Typical ligation complexes of Cu (II) by urea^37^ ligating can occur via the oxygen atom^36^ (Figure 6A) or nitrogen-based compounds^37^ (Figure 6B–E), such as octahedral complexes of ammonia and Cu^2+^ (Cu­(NH_3_)_4_Cl_2_)^61^ (Figure 6B); hydroxylated copper-ammonia complex—Cu^2+^ (NH_3_)_4_(OH)_2_ (Figure 6C); or, hydroxylated copper-urea complex—Cu^2+^[urea]_4_(OH)_2_ (Figure 6D). The hydroxylated Cu^2+^[urea])_4_(OH)_2_ complex has the capacity to form intermolecular extensive hydrogen bonding with a urea ligand from another complex (Figure 6E). Hydrogen bonding deactivates the surface group of the octahedral structure of the copper catalyst. Adsorption of the EPFR precursor (MCP), as demonstrated in the conventional model for EPFR formation on the catalyst, is therefore hindered or limited (Figure 6E), reducing the concentration of EPFRs that would normally have been formed in the absence of urea.

**Figure 6:**
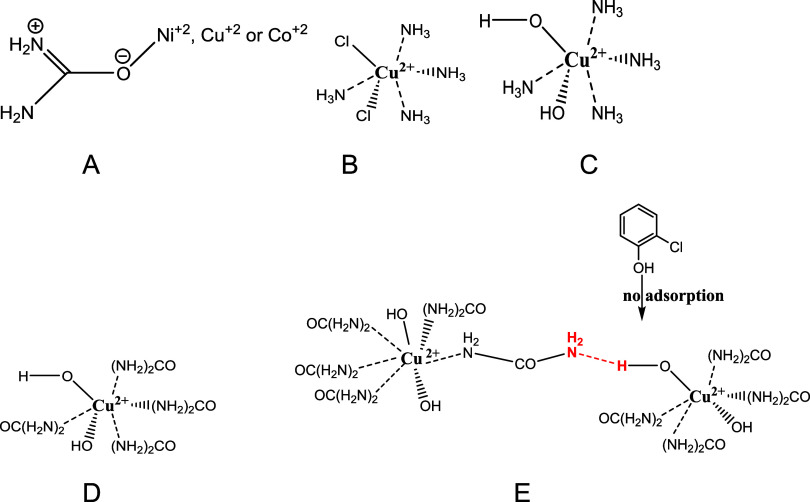
(A) Binding transition metals like Ni^2+^ to urea can occur through either oxygen (as in the structure shown) or (B–D) nitrogen (from sources like ammonia or urea, seen in copper complexes, as in the structure shown). (E) Complex features hydrogen bonding, which deactivates surface O–H groups, thereby reducing the rate of EPFRs formation.

Regardless of the class of compound, it is reported that a few functional groups with electronegative atoms, such as oxygen, nitrogen, and sulfur (electron-rich centers), act as ligand-chelators to form tetrahedral (or octahedral) complexes with transition metals.^62^ In particular, such centers are capable of coordinating with Cu­(II) contained in the catalyst. Apart from urea, water molecules also ligate well with Cu^2+^ in the matrix.^63^ Water has two lone pairs of electrons on its highly electronegative oxygen atom, analogous to that of nitrogen in urea, and can therefore form complexes with copper, which are stabilized by hydrogen bonding with neighboring urea and/or water molecules.^63^ Water and urea could therefore play a synergistic role (ref Figure S5) to bring about the enhanced reduction of EPFRs already generated in a system, as it has also been shown in this work (Section 3.2). Note that the presence of sulfates also limits the formation of EPFRs due to inhibition or poisoning of the transition metal active sites necessary for their formation.^64^

### 3.8 Proposed Mechanism for the Acceleration of the Decay of Already Formed MCP230 EPFRs

Ammonia is currently an effective reducing agent for selective catalytic reduction (SCR) of industrial NOx gases by transferring them (NO, NO_2_) into nitrogen. The release of ammonia from pyrolysis of urea, both in the solid state and in the solution with water, methanol, or ethanol in SCR reactors, is well established.^65–68^ The results of the present study for the acceleration of the decay of already formed EPFRs can also be related to previous literature studies concerning the interaction of ammonia with transition metal oxides, which are all active catalysts in SCR (as well as selective catalytic oxidation – SCO) processes.^68^

It is a commonly accepted fact that the reduction process occurs on the transition metal cationic center, Me^n+^ (Lewis sites), where both ammonia and NO could adsorb.^69,70^ More than one species is formed from ammonia over the numerous catalyst surfaces, and hydrazine is likely one such species. Looking at the possible mechanisms for ammonia oxidation to dinitrogen (SCO process), the intermediate formation of hydrazine as the first N–N bond-containing species seems indeed quite plausible.^69,70^ Ammonia can easily lose one hydrogen, giving rise to amide species (−NH_2_), which have been observed on SCR catalysts.^69,71^ The first step involves the activation of ammonia, and further interaction of the amide moiety (Me^(*n*–1)+^-NH_2_) with NO in the SCR process,^72^ referred in the literature as “amide-nitrosoamide” mechanism.^70,73^ It is assumed that a key reaction step is supposed to involve a radical coupling between the (Me^(*n*–1)+^-NH_2_) surface species (i.e., the amide moiety would act as a neutral radical), and with the radical molecule NO. It seems consequently very reasonable to suppose that such species (Me^(*n*–1)+^-NH_2_) tend to undergo a radical-like coupling, easily giving rise to hydrazine detected experimentally on different metal oxide surfaces^69,70^ , including CuOx in an early publication.^74^ Therefore, ammonia released from urea acts as a strong reducing agent, reduces Cu^2+^ to Cu^1+^ ^70^ and, for simplicity, is assumed to produce a surface-associated (^•^NH_2_)­s radical (reaction 1) and recombines itself in a termination reaction to form hydrazine, (N_2_H_4_)­g (reaction 2).

1 NH3+Cu+2→(NH2•)s+Cu+1+H+

2 2(NH2•)s→(N2H4)g

This active reductant (^•^NH_2_)­s reacts with NO at rates typical of gas-phase radical reactions to produce a relatively strongly bound H_2_NNO adduct that readily rearranges and decomposes to N_2_ and H_2_O.^75^ In contrast, (^•^NH_2_)­s reacts with O_2_ exceedingly slowly: the H_2_N-OO adduct is weakly bound and more readily falls apart than reacts to products.^75^ Consequently, (^•^NH_2_)­s can also be terminated with EPFRs (for simplicity, abbreviated as ^•^R, reaction 3) generated in our system. We hypothesize the acceleration of the decay of already formed EPFRs (abbreviated ^•^R) via recombination with surface-stabilized hydrazyl (^•^NH_2_)­s radicals

3 (NH2•)s+R•→(RNH2)g

In fact, (^•^NH_2_)­s are quenching EPFRs at the surface to form stable R-NH_2_ molecules released into the gas phase, thereby reducing the concentration of EPFRs (eq 3) in the mixture. Alternatively, in the presence of trace oxygen, Cu^1+^ can be easily oxidized back to Cu^2+^ (reaction 4)^43^

4 $$2{\mathrm{Cu}}^{+1}+2H^{+}+1/2O_{2}\to H_{2}O+{\mathrm{Cu}}^{+2}$$

Note that the system operates under a slow-diffusion regime in which the rate-limiting step of the overall chemical transformation is the diffusion of urea through the solid matrix. The reactions described in steps 2, 3, and 5 are fast coupling processes and therefore do not control the overall rate of EPFR decay in the presence of urea. If the diffusivity of urea is increased, then the rate of EPFR decay correspondingly rises. This effect was indeed observed when an aqueous urea solution was applied to solid EPFRs (Figures 2B and S5). In that case, the EPFR signal intensity decreased sharply (up to 97% reduction). One contributing factor is the substantially higher diffusion rate of urea in water toward the Cu sites, although the process still proceeds under diffusion-controlled conditions.

In other words, the dissolved urea and/or water molecules are ligating easily with Cu^2+^ by extrusion of adsorbed EPFRs on the copper site. It is worth noting from the XPS measurements (Figure 3a) that urea either replaces or removes the adsorbed MCP230 EPFRs from the CuO surface when it is added to the MCP230 EPFRs at room temperature. In this scenario, the “naked” EPFRs just disappear by mutual recombination (cross-coupling) reactions (reaction 5)­

5 R•+R•→R−R

Another scenario arises from the decomposition (hydrolysis) of urea in a water environment and in the presence of transition metals.^76^ A detailed mechanism is provided in Mavis and Akinc.^76^ Decomposition of urea is exceedingly slow in an aqueous solution in the absence of a catalyst (with a half-life of approximately 40 years at 25 °C).^77^ However, in the presence of urease, typically one of the most efficient enzymes, the half-life of urea decreases to about 20 ms at 25 °C.^77^ In both pathways, ammonia is released, and the second pathway, which is biologically relevant and likely leads to the radical-chain reactions 1–3, is favored. Indeed, the primary pathway for the release of ammonia on fertilized fields is through the urease-supported hydrolysis of urea. Ammonia may also interact with the transition metals abundant in the soil, and radical-chain reactions 1–3 may potentially lead to a decrease in the yields of EPFRs. It should also be noted that reactions 1 and 4, while being slow at room temperature, are similarly fast with increasing temperature^43,79^ and therefore do not limit the overall process.

It is also noteworthy that the urea mitigation/remediation effect, the reduction of the EPFRs concentration, is not without precedent.^78^ Urea has been reported^78^ to significantly reduce the amount of EPFRs in biochar from the pyrolysis of pure cellulose, and it is postulated that such biochar is good for agriculture because of reduced concentration of EPFRs and the increased nitrogen content in the biochar that are both good for plant growth. Significant EPFRs reduction in the biochars was observed after the addition of 40–50% urea compared to 10% in this work.

## 4 Conclusions

This study investigates the mitigating effect of urea on the formation of MCP230 EPFRs under controlled laboratory conditions. A strong inhibition of 2-MCP-EPFR formation was observed with increasing urea concentration, up to 10% (w/w) of the PM mass. The loss of Cu^2+^ activity toward EPFR formation in the presence of urea is likely due to the complexation of Cu^2+^ by urea (or by various decomposition products of urea, including ammonia, isocyanic acid, hydrazine, etc.), as evidenced by EPR, XPS, and XAS analyses. Formation of these complexes and intermolecular hydrogen bondings prevents the adsorption of EPFR precursor molecules onto surface-bound hydroxyl groups.

Alternatively, it is proposed that, in addition to urea–copper complex formation, the degradation of pre-existing EPFRs, generated either in the laboratory or present in field samples, is accelerated with increasing addition of urea, both in solid form and, more effectively, in aqueous solution. The observed remediation effect, i.e., the reduction of the EPFR concentration in complex systems, is hypothesized to result from enhanced radical coupling reactions between EPFRs and surface-stabilized *radical-like species* such as amide groups (Me^(*n*–1)+^–NH_2_), leading to the formation of organic amides at elevated temperatures. Additionally, we hypothesized that the extrusion of adsorbed EPFRs from copper sites due to the strong ligating of urea to copper may facilitate their disappearance via mutual recombination (cross-coupling) reactions, even at room temperature.

Additionally, we highlight the need to include complementary techniques in future research to better explore the nature of Cu^2+^ complexes with urea as well as possible complexes formed with urea decomposition products such as ammonia, isocyanic acid, and hydrazine.

## Supplementary Material



## Notes

Full experimental procedures for the gas-phase exposure chamber and combustion reactor are detailed herein, alongside measurement protocols for XANES, XPS, and EPR spectroscopy. This includes the application of EasySpin code for radical characterization and the methodology used to assess how urea alteration modulates EPFR formation and decay kinetics in both laboratory and field-derived samples (PDF)
