# Supplementary material for: Urea Inhibits the Formation of Environmentally Persistent Free Radicals as well as Accelerates Their Decay
Source: ACS Omega. 2026 Apr 30;11(18):26635–45. doi: 10.1021/acsomega.5c13197 (PMC13177020; doi:10.1021/acsomega.5c13197)
Supplement: Supplementary file 1 [file ao5c13197_si_001.pdf]

SUPPORTING INFORMATION

Urea Inhibits the Formation of Environmentally Persistent Free Radicals (EPFRs) as well as Accelerates Their Decay.

Divine Nde[1], Fox Foley[2], Orhan Kizilkaya[3], Myron Lard[4], Jennifer Richmond-Bryant[4,5], Lavrent Khachatryan[1*], Slawomir Lomnicki [6], Phillip Sprunger[2*], Stephania A. Cormier [7]

[1] Department of Chemistry, Louisiana State University, Baton Rouge, Louisiana 70803, USA

[2] Department of Physics and Astronomy, Louisiana State University, Baton Rouge, Louisiana 70803, USA

[3] Center for Advanced Microstructures and Devices, Louisiana State University, Baton Rouge, LA 70806, USA

[4].Department of Forestry and Environmental Resources, North Carolina State University, Raleigh, NC, 27695, USA

[5].Center for Geospatial Analytics, North Carolina State University, Raleigh, NC, 27695, USA

[6] Department of Environmental Sciences, Louisiana State University, Baton Rouge, Louisiana 70803, USA

[7] Department of Biological Sciences, LSU Superfund Research Program and Pennington Biomedical Research Center, Baton Rouge, Louisiana 70808, USA

1. Gas-phase Exposure chamber for generation of MCP230 EPFRs: A scheme for generation of EPFRs from simple aromatics such as MCP, DCB is represented in **Figure S1**. It is a simple vacuum line consisting of a container with a precursor (dosing port), as short as possible transfer vacuum line, thermoelectric furnace, and a vacuum chamber for the solid catalyst, 5%CuO.SiO_2 nano particles [1]. Depending on the amount of solid sample either large ball-shape dosing chamber was used or a large EPR tube (o.d. = 10mm) for small amounts (less than 30mg). The vacuum system (including a vacuum pump, pressure gauge and cold trap) performed a dual function by drying and cleaning the catalyst of any contaminants prior to the adsorption experiments.

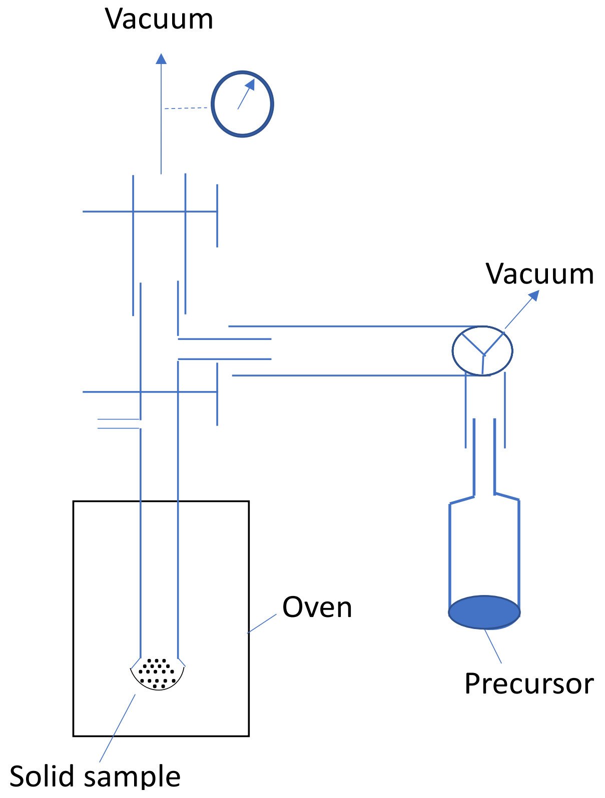

Figure S1. Gas phase exposure chamber for generation of EPFRs from simple aromatics such as MCP, DCB etc.

For the experiments, about 35-40mg of the catalyst were weighed into the EPR larger tube (dosing chamber) which was then attached to the experimental set-up as shown in **Figure S1**. The MCP solution was subjected to deaeration (degassing) from dissolved air by immersing the container in liquid nitrogen and evacuating the air under vacuum after freezing/thawing cycle repeated three times. The pressure of the system under vacuum was 0.01torr. As the vacuum was established the catalyst was heated at 120 °C for 15min and equilibrated the temperature in the chamber at 230 °C for 30min. This was followed by the adsorption process in which the vacuum lines were closed to give one direct line between the MCP and the catalyst. Exposure of MCP to the catalyst in the exposure chamber was done at 5min intervals several times. The vane was opened after each 5 min exposure to refresh the

vacuum up to 0.01 torr and repeat another exposure cycle as needed. After exposure, the system was allowed to cool down to 50 °C and then taken for EPR measurements.

2. Two stage combustion reactor (TSCR): A special, dual zone reactor, TSCR, (**Figure S2**) is used to generate combustion derived EPFRs. This reactor allows us to generate increasingly complex EPFRs, while controlling radical and metal oxides (Fe_2O_3, CuO, NiO etc.) concentrations. The design of the TSCR reactor is based on our published studies to investigate the sooting phenomenon of 1-methylnaphtalene (1-MN) in the gas phase. [2, 3]

Briefly, Zone 1 of the dual zone reactor generates entrained metal oxide nanoparticles from the high-temperature oxidation of reverse micelle suspension of metal oleate in hexane. The Zone 1 reactor is

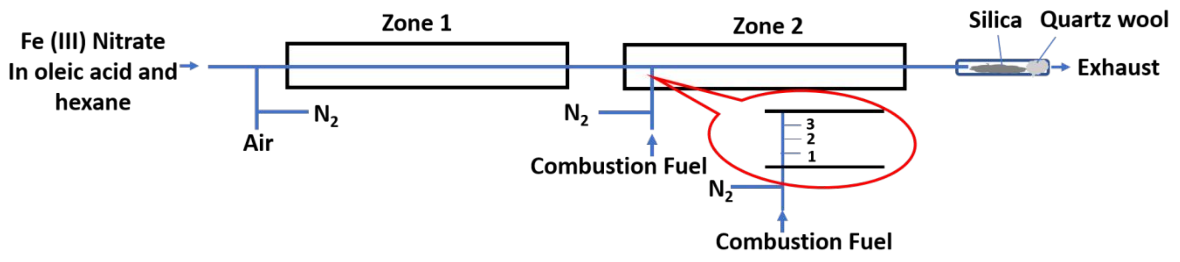

Figure S2. Schematic view of TSCR. The injection port temperatures at different locations were (1) 400ºC at 1 cm, 700ºC at 2 cm (2) and 940ºC at 3 cm (3) from the outside wall of the furnace and at 970ºC in the center of the zone 2.

maintained at 700°C with a gas-phase residence time of 60 seconds. The Zone 1 reactor interfaces with a Zone 2 combustor, and gases with particles from Zone 1 are transported in a gas phase passing through a cooling section to ensure nucleation of the metal oxide nanoparticles before reaching Zone 2.

The Zone 2 reactor is maintained at 970ºC with a gas-phase residence time of 1 second at flow rate of the carrier gas (54 cc/min). This flow rate ensured the introduction of the fuel to the Zone 2 reactor by syringe pump with no back flow of the gases into Zone 1. The combustion fuel is delivered at the beginning of the Zone 2 through a side port arm, **Figure S2**. The control of EPFRs concentration is achieved by changing the parametric combustion conditions through alteration of the cross-sectional location of fuel injection point in Zone 2; the highlighted in red cycle presents the cross-sectional temperature gradient in the fuel injection area and injection locations.

The fuel is comprised of chlorinated phenols and polyaromatic hydrocarbons (PAHs): 1- Methylnaphtalene (1-MN) + 1-Monochlorophenol (1-MCP, 30%v/v) + Anthracene (5.3 mg / 30 ml). The EPFRs generated in the TSCR are collected thermophoretically on Cab-o-Sil matrix at the exit of Zone 2, Figure S2. The sample yields from TSCR are dependent on fuel-to-air equivalence ratio and temperature in Zone 2 and are a function of introduced metal concentration. Approximately 10 mg of sample is generated in 30 min. The EPFRs containing samples collected are dried in a vacuum oven for 50 min and then taken for EPR measurements.

A typical EPR spectra of the samples generated by the combustion reactor are characterized by a relatively broad (ΔHp-p ~6-10 Gauss), asymmetric signal with a g value of 2.0028-2.0032. [2, 3]

3. XANES and XPS of interactions between catalysts & urea

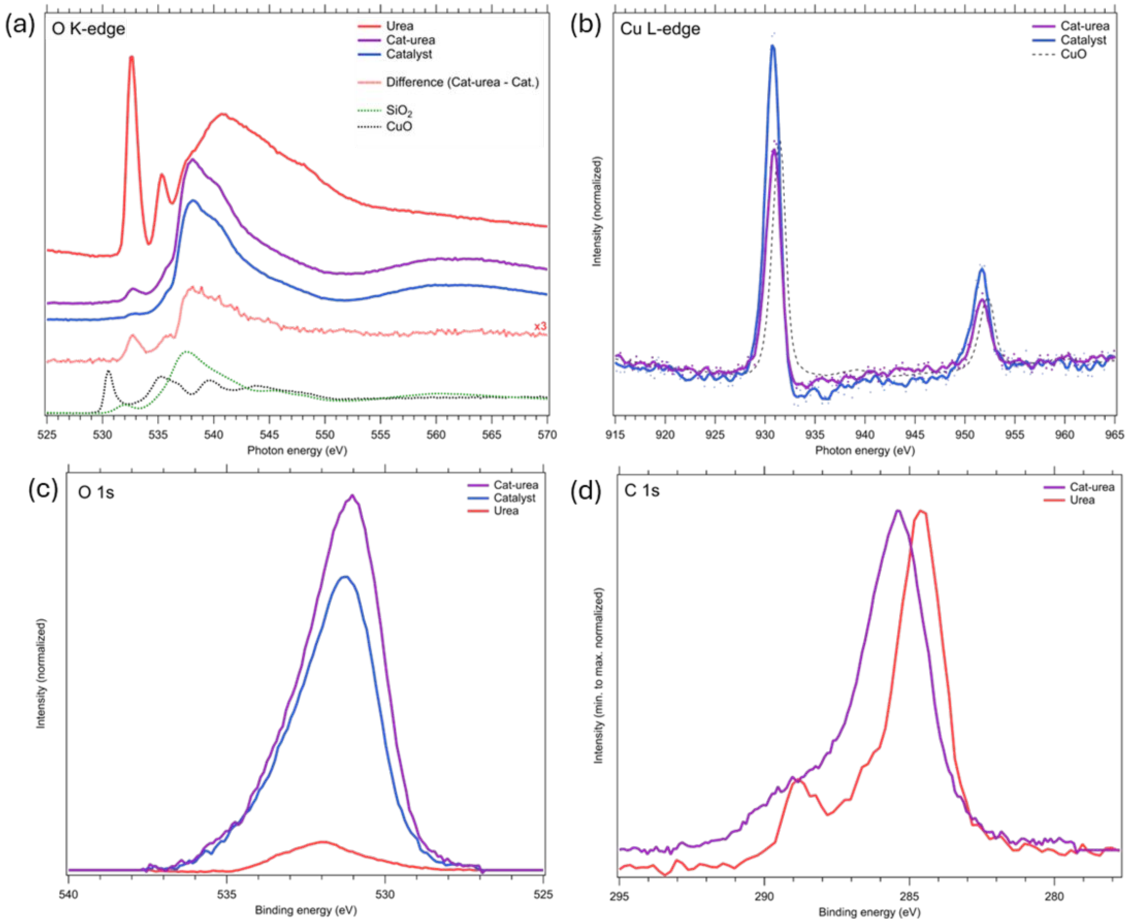

Figure S3. (a) O K-edge selected standards and samples. Difference spectrum (cat-urea – catalyst) shows two prominent peaks at 533 and 537.5 eV (b) Cu L-edge of CuO standard, catalyst, and cat-urea shows attenuation of intensity from catalyst to cat-urea. (c) O 1s of urea, catalyst, and cat-urea showing increasing O content in the same order (d) C 1s XPS spectra of cat-urea and urea. Samples were not annealed beforehand and thus contain some adventitious carbon (advC), which accounts for the primary C 1s peak around 285 eV. Urea shows a prominent C=O peak at 289 eV, which appears as a shoulder in the cat-urea spectrum: indication of the presence of -CO bonds absent in advC.

3.1 The lifetime of MCP230 EPFRs [1]

The lifetime of EPFRs is specified as a time, (t) when the initial concentration of EPFRs may drop 1/e times (where the (e) is the base of natural logarithm). For pseudo-1st order decay reactions this lifetime, $t_{1/e} = 1/\mathbf{k}$, where **k** is the rate constant for the decay reaction of EPFRs (here for pseudo first order reaction), and can be determined experimentally by using standard equation (Eq.S1),

log C/Co = -k/2.3 x t **(Eq.S1)**

where Co and C are initial and current concentration of EPFRs, respectively and k is the pseudo unimolecular reaction rate coefficient. The intensity of radicals is measured by EPR technique.

Fresh and aged EPFRs from MCP 230: The secondary reactions occurring on the surface of the matrix (CuO/SiO_2) in mostly aerobic conditions dramatically influence on the nature of EPFRs leading to acceleration of the decay process; this results in changing of the average g value or the width of EPR spectra for the mixture of EPFRs. A typical profile of aging is shown in **Figure S4;** the lifetime of fresh radicals during 1st hour measurements is ~ 91min at g = 2.0042, and the g value drops to 2.0036 after further long-term measurements (7 days). Therefore, the 1/e time is highly sensitive to experimental conditions, particularly the vacuum quality in the EPR tube, and can vary significantly depending on the aging conditions.

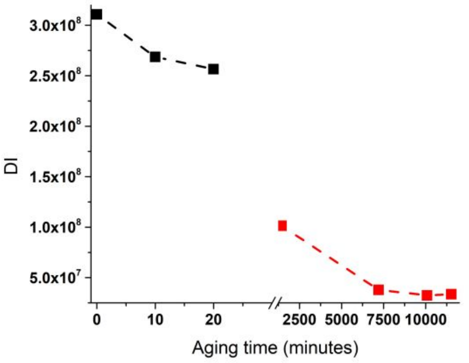

Figure S4. Aging phenomenon of MCP230 EPFRs in an EPR tube under vacuum. The experimental data points are averages obtained from 2 or 3 trials. DI-Double integration of the EPR spectra; $t_{1/e}$ fresh =91min, g = 2.0042; black squares; $t_{1/e}$ aged = 208 hrs, g = 2.0036; red squares.

3.2. Solid urea, Urea aqueous solution and water only accelerate the decay of EPFRs generated from different sources:

The application of DI water, solid urea to the MCP EPFRs generated from exposure chamber at 230 C (**Figure S5**, A, B**,** respectively**)** plays a critical role in accelerating the decay of already formed radicals. A significant reduction with application of 10% aqueous urea solution (a markedly higher abatement rate of 97%, the final graph in **Figure S5**, A) indicates a synergy between water (an abatement of 80%, A) and solid urea (82 %, B). Similar phenomenon has been observed for EPFRs generated from combustion reactor (**Figure S5**, C).

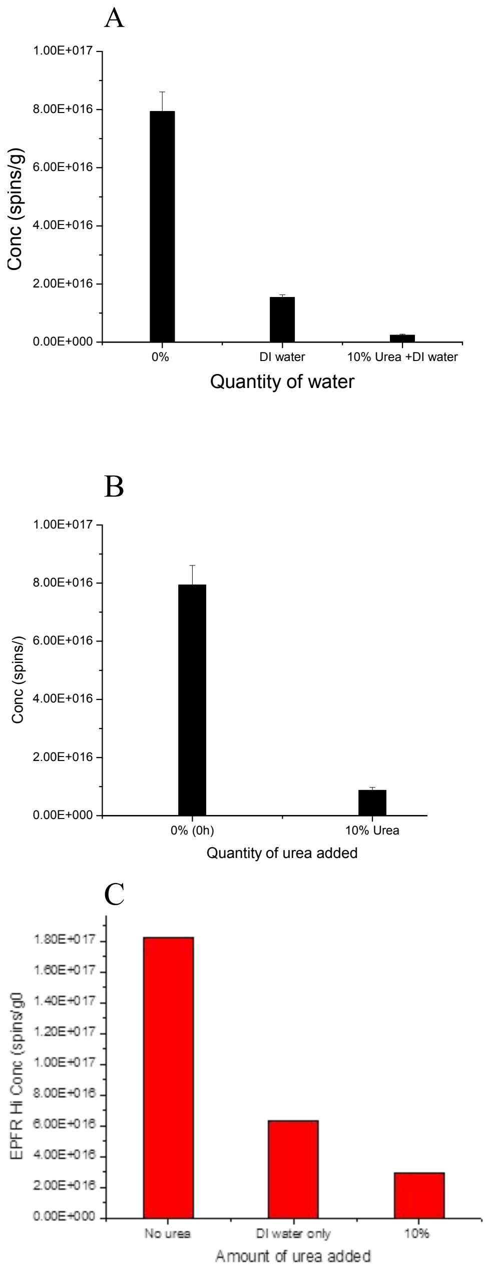

Figure S5. Decay bars for EPFRs generated from MCP under laboratory conditions following treatment with (A) pure water and (B) solid urea. Pure water produced an EPFR abatement of 80% (A), compared with 82% following the addition of solid urea (B). In contrast, a 10% aqueous urea solution resulted in a markedly higher abatement rate of 97% (final bar in A). The same scenario has been observed on the acceleration of the decay of EPFRs from combustion reactor, (C).

3.2.1. Remediation of contaminated soils treated with aqueous urea solution.

The mitigation process, involving the prevention of EPFRs formation or acceleration of their decay, is pivotal in reducing the environmental risks associated with EPFRs. The effect of urea $(NH_2)_2C=O$) mitigator has been shown for the samples from Colfax. A typical content of the sample from Colfax site using ICP-OES analysis is presented in Table S1. The composition of soil collected from Superfund sites is complex (Table S1) and among the inorganic/organic contents contain also EPFRs, **Figure S6** (black line detected at room temperature). Urea Accelerates the decay of f-EPFRs (EPFRs from the field) from the waste deposited in Superfund sites, Colfax. A drop of EPR signal intensity ~ 20 - 25 % has been detected at impregnation of Colfax sample (abbreviated 2B) into 10% urea solution (red line).

Table S1. ICP-OES analysis of two samples from Colfax superfund site

Sample	Calcium (mg/kg)	Copper	Iron	Magnesium	Manganese	Sodium	Zink	Carbon
1,2-mi S*	772.3	0.0	5330.7	365.7	203.7	44.2	213.6	24030.0
9.0-mi SSE*	1735.1	8.7	14319.4	5153.1	317.4	145.3	213.6	13250.0

* Indicates the distance in miles and direction from the thermal treatment plant in Colfax

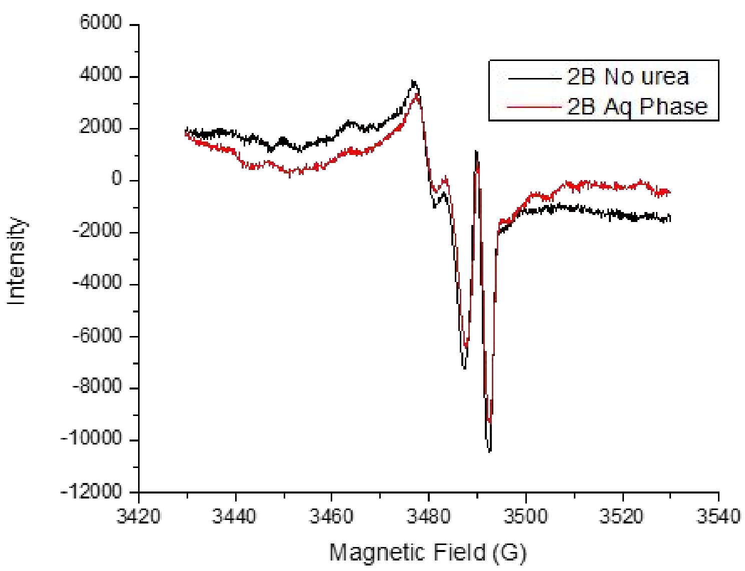

Figure S6. Urea Accelerates the decay of f-EPFRs (EPFRs from the field) from the deposited waste in Superfund sites, Colfax. A drop of EPR signal intensity ~ 20 -25 % has been detected at impregnation of Colfax sample (abbreviated 2B) into 10% urea solution (red line).

3.3. Paramagnetic Species Formed During MCP230 EPFR Generation in the Presence of Urea. A singlet spectrum of MCP230 EPFRs was detected in presence of urea in the matrix, spectrum 1, **Figure S7, A**. The intensity of fresh spectrum 1**, Figure S7, A** with a g value of 2.0040 drops essentially after 2 days aging at apparent g value of 2.0034, spectrum 2.

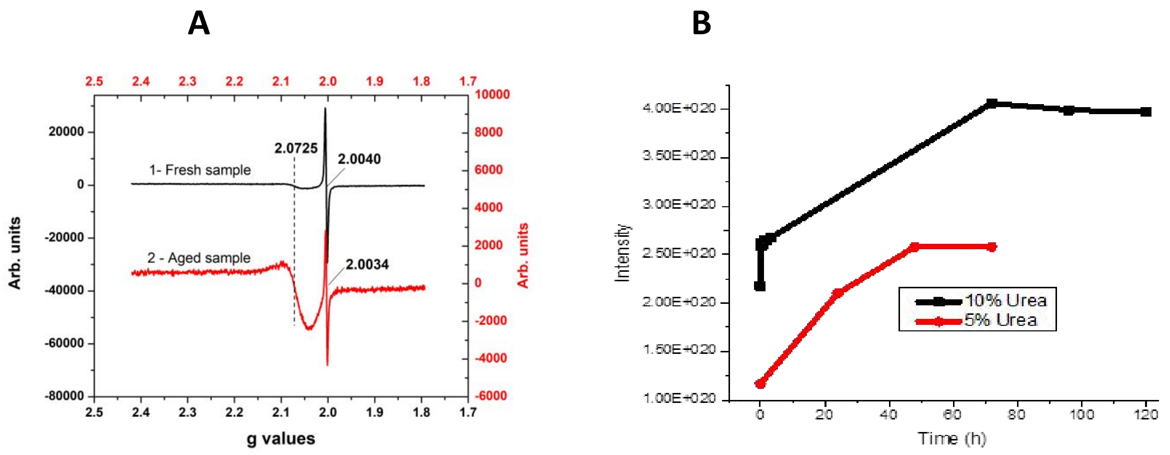

Figure S7. (**A**) - The EPR spectra of fresh MCP230 EPFRs (**spectrum 1**) and aged for 2 days in vacuum (**spectrum 2**) in presence of urea (10 %, w). (**B)** - The EPR spectral intensity of Cu^{+2} (**g_{iso}** (Cu^{+2}) = 2.0750) shows a dynamic increase (in arbitrary units) in presence of 5% and 10% urea over time.

3.3.1. g and ΔH p-p values for MCP230 EPFRs without and with urea in the matrix:

A detectable decrease in g and ΔHp-p values has been observed in presence of urea indicating a change of the matrix environment, **Figure S8**.

A

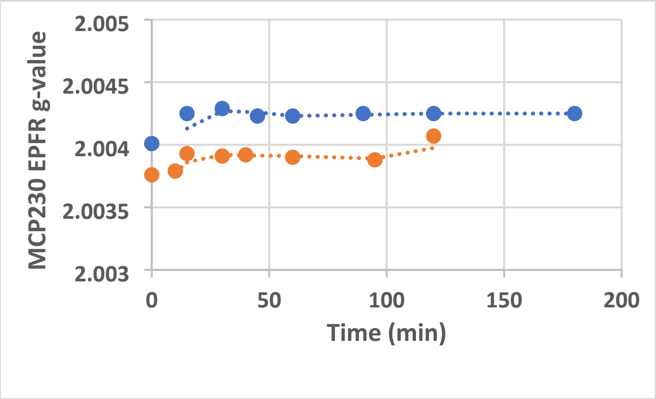

B

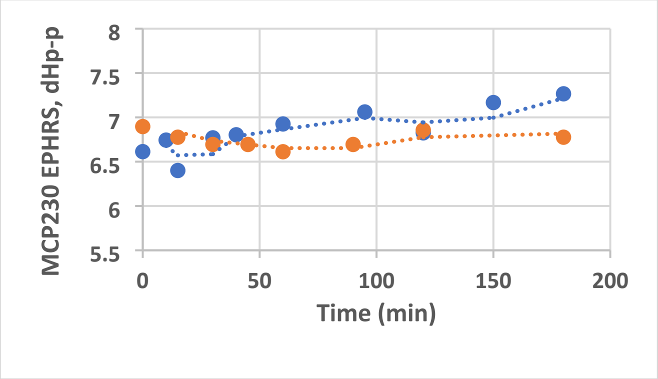

Figure S8. g-values (A) and dHp in gausses (B) for MCP230 EPFRs without urea (blue) and with urea (orange).

4. Easy Spin simulation code [4].

```
[B_G,spc] = eprload('10%urea_ 5000G on 021422 2mW.spc'); EPR experimental spectrum
B = B_G/10; transfer of gausses to milli-tesla
plot(B,spc);
% Set up experiment
Exp.mwFreq = 9.753934;
Exp.Range = [86.65 586.65]; % mT could be [min(B) max(B)].
%Exp.nPoints = numel(spc);
% Set up spin system with starting parameters
Cu.Nucs = 'Cu';
```

```
Cu.A = [52 461]; % in MHz
Cu.g = [2.06 2.06 2.26];% was[2.0882 2.0882 2.3900];
Cu.lwpp = [0 7]; % in mT, Lorentian if in skobkax
Cu.gStrain = [1 1 1]*0.02;
% Set up Vary structure with all parameters that can be varied
CuVary.g = [1 1 1]*0.02;% was *0.03
CuVary.gStrain = [1 1 1]*0.02;%was 0.2
CuVary.A = [1 1]*10; % in MHz
esfit(@pepper,spc,Cu,CuVary,Exp);
```

5. A conventional mechanism for formation of MCP230 EPFRs [1,5]

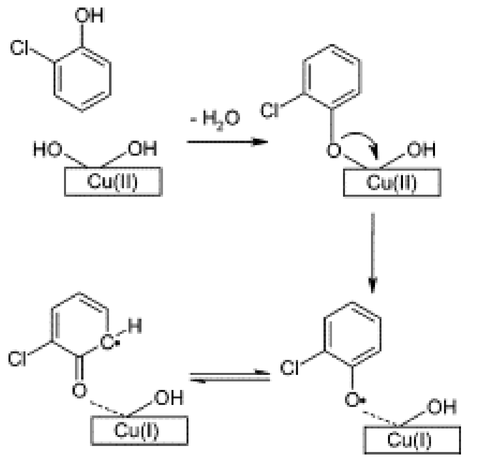

Scheme S1: A conventional mechanism for the formation of persistent free radicals, EPFRs on copper (II) oxide/silica surface under adsorption of 2-monochlorophenol [5].

References

(1) Lomnicki, S.; Truong, H.; Vejerano, E.; Dellinger, B. Copper oxide-based model of persistent free radical formation on combustion-derived particulate matter. *Environmental science & technology* **2008**, *42* (13), 4982-4988.

(2) Herring, M. P.; Potter, P. M.; Wu, H. Y.; Lomnicki, S.; Dellinger, B. FeO nanoparticle mediated molecular growth and soot inception from the oxidative pyrolysis of 1-methylnaphthalene. *P Combust Inst* **2013**, *34*, 1749-1757. DOI: 10.1016/j.proci.2012.07.057.

(3) Aryal, A.; Noël, A.; Khachatryan, L.; Cormier, S. A.; Chowdhury, P. H.; Penn, A.; Dugas, T. R.; Harmon, A. C. Environmentally persistent free radicals: Methods for combustion generation, whole-body inhalation and assessing cardiopulmonary consequences. *Environmental Pollution* **2023**, *334*, 122183.

(4) Stoll, S.; Schweiger, A. EasySpin, a comprehensive software package for spectral simulation and analysis in EPR. *J Magn Reson* **2006**, *178* (1), 42-55. DOI: 10.1016/j.jmr.2005.08.013.

(5) Alderman, S. L., Farquar, G. R., Poliakoff, E. D., Dellinger, B. An infrared and X-ray spectroscopic study of the reactions of 2-chlorophenol, 1,2-dichlorobenzene, and chlorobenzene with model CuO/silica fly ash surfaces. *2005* **2005**, *39*, 7396-7401.
